# Upregulation of mir-1199-5p is associated with reduced type 2 5-α reductase expression in benign prostatic hyperplasia

**DOI:** 10.1186/s12894-022-01121-5

**Published:** 2022-11-07

**Authors:** Zhanliang Liu, Zhemin Lin, Fang Cao, Mingxin Jiang, Song jin, Yun Cui, YN Niu

**Affiliations:** 1 Beijing Shijitan Hospital, Capital Medical University, 100038 Beijing, China; 2 Department of Urology, Beijing Chaoyang Hospital, Capital Medical University, 100016 Beijing, China; 3 Department of Urology, Beijing Tsinghua Changgung Hospital, Tsinghua University, 102218 Beijing, China

**Keywords:** 5-α reductase, Benign prostatic hyperplasia, miR-1199-5p

## Abstract

**Background:**

5-α reductase inhibitors (5-ARIs) are first-line drugs for managing benign prostatic hyperplasia (BPH). Unfortunately, some patients do not respond to 5-ARI therapy and may even show worsening symptoms. The decreased expression of steroid 5-α reductase type 2(SRD5A2) in BPH tissues may explain the failure of 5-ARI therapy, however, the mechanisms underlying SRD5A2 decreased remained unelucidated.

**Objectives:**

To investigate microRNA-mediated regulation of the expression of SRD5A2 resulting in 5-ARI therapy failure.

**Materials and methods:**

The expression of SRD5A2 and microRNAs in BPH tissues and prostate cells were detected by immunohistochemistry, western blotting, and quantitative real-time PCR. Dual-luciferase reporter assay was performed to confirm that microRNA directly combine to SRD5A2 mRNA. The apoptosis of prostatic cells was detected by flow cytometry.

**Results:**

SRD5A2 expression was variable; it was negative, weak, and strong in 13.6%, 28.8%, and 57.6% of BPH tissues respectively. The normal human prostatic epithelial cell line RWPE-1 strongly expressed SRD5A2, whereas the immortalized human prostatic epithelial cell line BPH-1 weakly expressed SRD5A2. miR-1199-5p expression was remarkably higher in BPH-1 than in RWPE-1 cells(P<0.001), and miR-1199-5p expression was significantly upregulated in BPH tissues with negative SRD5A2 expression than those with positive SRD5A2 expression. Transfection of miR-1199-5p mimics in RWPE-1 cells led to a marked decrease in SRD5A2 expression, whereas miR-1199-5p inhibitor increased SRD5A2 expression in BPH-1 cells. Dual-luciferase reporter assay showed that miR-1199-5p could bind the 3′untranslated region of SRD5A2 mRNA. miR-1199-5p also decreased the RWPE-1 sensibility to finasteride, an inhibitor of SRD5A2.

**Conclusion:**

Our results show that SRD5A2 expression varies in BPH tissues and miR-1199-5p might be one of the several factors contributing to differential SRD5A2 expression in BPH patients.

**Supplementary Information:**

The online version contains supplementary material available at 10.1186/s12894-022-01121-5.

## Introduction

Benign prostate hyperplasia (BPH) is histologically characterized by the aberrant proliferation of epithelial and stromal cells in the prostatic transition zone 1. BPH incidence increases with age; approximately 50% of men in their 50s and over 80% in their 80s show pathological manifestations of BPH 2, 3. The overgrowth character of the prostate indicates that approximately 25% of men will develop BPH clinical symptoms in their life 4. BPH deteriorates the life quality of older men because of lower urinary tract symptoms (LUTSs), including urination and retention 1, 5.

Watchful waiting, drug therapy, and surgery are currently used as BPH therapies 6. Most patients receive medical therapy, mainly including α-adrenergic blockers and 5-α reductase inhibitors (5-ARIs) when LUTSs first occur 7. However, only 5-ARIs effectively reduce the prostate size by approximately 20–30% in 4–6 months 8. 5-ARIs target 5-α reductase (SRD5A) and block the transformation of testosterone (T) to dihydrotestosterone (DHT), which has a higher affinity to androgen receptors (ARs). Reduced DHT concentration in the prostate induces apoptosis and necrosis of AR-dependent cells and eventually reduces the prostate size 8, 9. Finasteride and dutasteride are the two primary 5-ARIs drugs that target different 5-AR isotypes. Finasteride specifically inhibits SRD5A2, mainly expressed in the prostate 10, whereas dutasteride inhibits both 5-α reductase 1 and SRD5A2; both these inhibitors show similar therapeutic efficacy.

Although 5-ARIs were considered the first-line therapy in BPH management, approximately 30% of patients showed no improvements after 5-ARI therapy, suffered worsened symptoms, and eventually required surgery 8, 11. Currently, little is understood about the mechanisms of 5-ARI treatment failure, making it impossible to predict the effectiveness of 5-ARI therapy on an individual. Thus, some patients undergo ineffective long-term treatment with associated adverse effects and unnecessary expenditure. Therefore, exploring the mechanisms associated with resistance to 5-ARI therapy is especially important.

Previous studies have reported significant variability in SRD5A2 protein expression in BPH samples, and 10 − 36.5% of BPH samples did not express SRD5A2 protein, corresponding to the proportion of patients resistant to ARIs 6, 8, 12. These findings suggest that decreased SRD5A2 expression in BPH tissues may be associated with 5-ARI therapy failure. Therefore, SRD5A2 may be a potential molecular marker for assessing responsiveness to 5-ARIs. However, the mechanisms underlying the suppression of SRD5A2 expression in prostate tissues are not fully understood.

miRNAs are extremely conserved short non-coding RNAs ranging from 19 to22 nucleotides, they regulate protein expression by binding to specific mRNA sequences 13. Like DNA methylation and histone modification, miRNA plays critical epigenetic roles in initiating and progressing many diseases 14, 15. A single miRNA can target several mRNAs to affect the expression of many genes 16. miR-1199-5p, located on chromosome 19p13.12, is an epithelial-mesenchymal transition (EMT)-regulatory miRNA that inhibits EMT and tumor cell invasion 17. Here, we demonstrate the decreased expression of SRD5A2 protein in 40.6% of the prostate samples and investigate association between miR-1199-5p and decreased SRD5A2 expression in BPH.

## Materials and methods

### Patients

Fifty-nine BPH specimens of transition zone were collected from patients undergoing transurethral resection of the prostate at the Beijing Chaoyang Hospital, Capital Medical University. Patient characteristics are shown in Table 1. All human specimens were acquired under the approval of the Institutional Review Board of Beijing Chaoyang Hospital, Capital Medical University (2017-KE-6, Beijing, China). The study was conducted in accordance with the World Medical Association Declaration of Helsinki, and written informed consent was obtained from each patient. The mean age of the BPH patients was 70 years, ranging from 56 − 91 years. Before being evaluated for SRD5A2 expression, the specimens were paraffin-embedded and confirmed to be BPH and non-cancerous through routine histological analysis by pathologists.

### Immunohistochemical analysis of SRD5A2

Immunohistochemistry (IHC) was performed as previously described by Lin et al. 9. Briefly, specimen sections were incubated with anti-SRD5A2 antibody (Novus Biological Inc., Centennial, CO, USA, NBP1-46510) following the manufacturer’s recommendations at a concentration of 1:1500. Negative controls were used throughout the immunostaining protocol. Three representative areas from each sample were randomly selected under 40× magnification to assess immunoreactivity by two genitourinary pathologists. A hundred cells selected randomly from the epithelium were manually counted from each representative section. Each cell was scored on a 0–3 scale according to the intensity of the staining. Then, a visual score was generated for each sample, ranging from 0 to 300. A score of 0–100 was defined as weak expression, and a score of 101–300 as strong expression.

### Cells and culture condition

The immortalized human prostatic epithelial cell line BPH-1 and HEK293T were obtained from the Cell Resource Center, Institute of Basic Medical Sciences, Chinese Academy of Medical Sciences (Beijing, China). Normal human prostatic epithelial cell line RWPE-1 was acquired from Shanghai Zhong Qiao Xin Zhou Biotechnology Co.,Ltd. BPH-1 cells were cultured in RPMI 1640 medium (Gibco, Rockville, MD, USA) supplemented with 10% fetal bovine serum (FBS; Gibco, Melbourne, Australia) and 1% penicillin -streptomycin (HyClone, Logan, UT, USA). 293T cells were cultured in Dulbecco’s Modified Eagle Medium (Gibco, Rockville, MD, USA) supplemented with 10% FBS and 1% penicililin -streptomycin. RWPE-1 cells were cultured in Keratinocyte Medium with a keratinocyte growth supplement. Cells were incubated at 37℃ with 5% CO2. All in vitro experiments were repeated three times.

### RNA extraction and quantitative real-time PCR (qRT-PC

**R)**

Total RNA was extracted using TRIzol reagent (Invitrogen Inc.,Carlsbad, CA, USA). The quality and quantity of extracted RNA were assessed using NanoDrop (Thermo Fisher Scientific, Waltham, MA, USA). Reverse transcriptase was used to produce the first-strand complementary DNA (TIANGEN, Beijing, China) according to the manufacturer’s instructions. miRNA Real-Time PCR Assay kit was used to detect miRNA expression levels (TransGen, Beijing, China). mRNA expression was normalized to GAPDH expression, whereas miRNA was normalized to U6 expression. The relative RNA expression was calculated using the delta/delta CT method. Specific primers are shown in Table S1.

### Cell transfection

miR-1199-5p mimics, miR-100-5p inhibitor, and miRNA negative control (miR-NC) were synthesized by Suzhou GenePharma Co., Ltd. (Suzhou, China) and transfected into cells using siRNAmate reagent (Suzhou GenePharma Co., Ltd., China) according to the manufacturer’s instructions. Total RNA was extracted for qRT-PCR after transfection for 24 h, and the protein was extracted for western blotting after transfection for 48 h.

### Western blotting

Cells were lysed for extracting total protein with RIPA buffer containing a mixture of protease and phosphatase inhibitors. The protein concentration was determined using the Pierce™ BCA protein assay reagent (Thermo, Rockford, USA). And proteins were separated by 10% sodium dodecyl sulfate polyacrylamide gel electrophoresis (SDS-PAGE). The protein bands were then electroblotted onto poly (vinylidene fluoride) (PVDF) membranes (Merck KGaA, Darmstadt, Germany). The membranes were blocked and cut according to the protein molecular weight, and then the blots were incubated with anti-SRD5A2(1:1000) or anti-GAPDH (1:2000) primary antibodies at 4 ℃ overnight. Subsequently, the PVDF membranes were washed three times with TBST and then incubated with horseradish peroxidase-conjugated secondary antibodies (1:2000; Abcam, Cambridge, UK) at room temperature for 1 h. Then, the membranes were washed again three times for 5 min each with TBST. Enhanced chemiluminescence (ECL) western blotting substrate was used for visualization and detection.

### Dual-luciferase reporter assay

For the dual-luciferase reporter assay, 293T cells were seeded in 24-well plates (50 000 per well) and co-transfected with a PGL-3 control plasmid, wild-type or mutant SRD5A2 3ʹUTR vector, and miR-1199-5p mimics or control (Suzhou GenePharma Co., Ltd, Suzhou, China). After 24 h, the cells were harvested, and Firefly/Renilla luciferase activities were analyzed using the dual-luciferase reporter assay kit according to the manufacturer’s protocol (Promega, Madison, WI, USA).

### Flow cytometry analysis assay

Annexin V-FITC and PI Detection Kit (BD Biosciences, NJ, USA) was used to stain RWPE-1 and BPH-1 cells to determine cell apoptosis. RWPE-1 or BPH-1 cells at a density of 2 × 10^5^ were treated with finasteride/DMSO and mimics/inhibitors. They were then harvested, resuspended in 100µL flow cytometry binding buffer, and then stained with 5µL Annexin V/FITC and 5µL PI following the manufacturer’s instructions. Apoptosis was determined using flow cytometry (BD FACSCanto™ II, NJ, USA).

### Statistical analysis

All statistical analyses were performed using the SPSS 23 software (SPSS, Chicago). Comparisons between datasets from qRT-PCR, western blotting, and fluorescence analysis were carried out using Student’s t-test. All tests were two-tailed, and P < 0.05 was considered statistically significant.

**Table 1:** Principal cohort characteristics (n = 59)

Variables	Mean	Standard deviation	Min	Max
Age, years	70.5424	7.43552	56	91
IPSS	22.5333	6.94676	12	33
PSA, ng/ml	6.489	6.59175	0.21	34.43
BMI	23.7269	2.68572	17.99	29.74
TPV, ml	75.9058	29.08218	24.52	146.89

IPSS, international prostate symptom score; PSA, prostate-specific antigen; TPV, total prostate volume

## Results

### SRD5A2 expression varies in different BPH tissues and cells

**Fig. 1:**
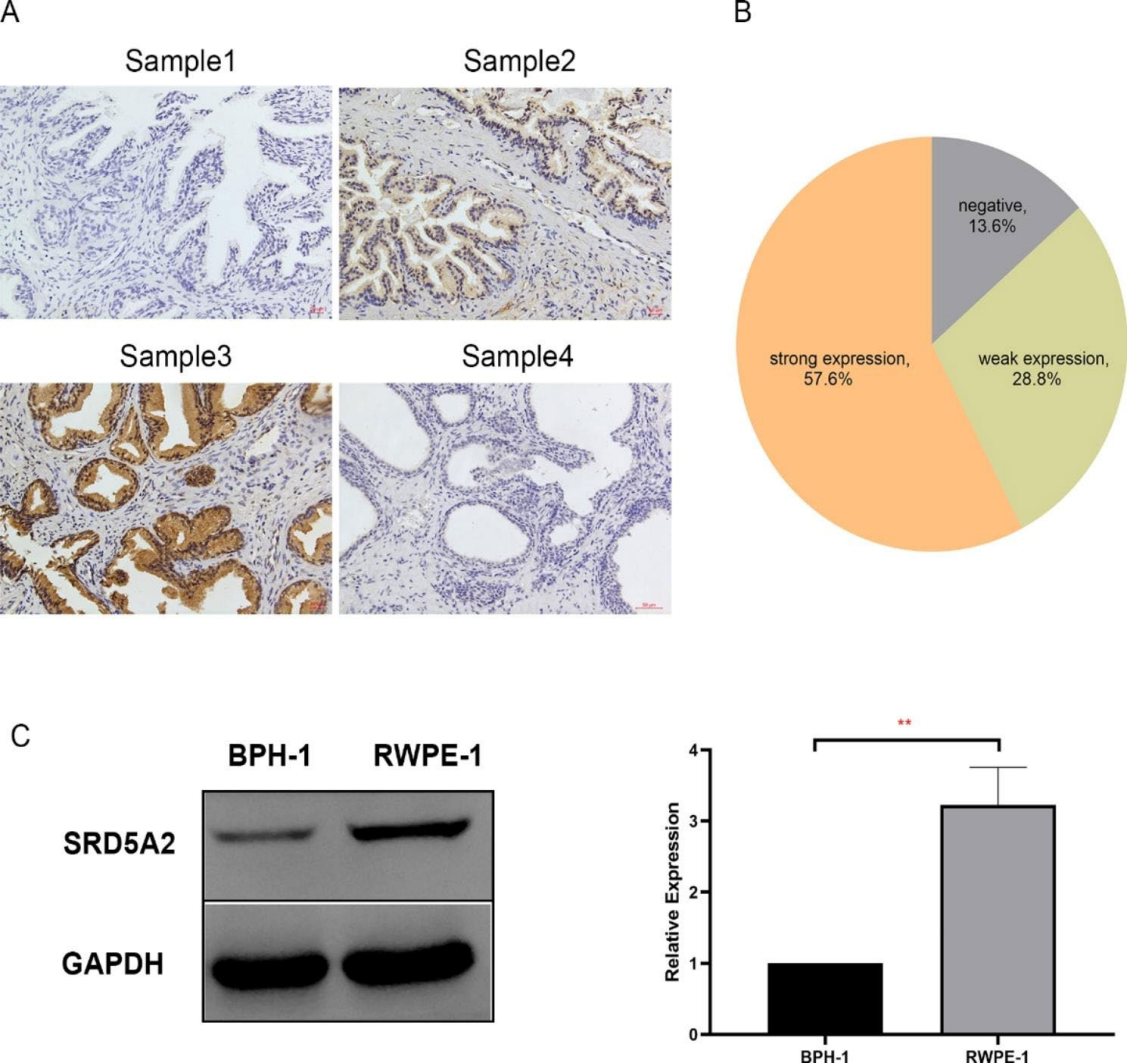
Diversity of SRD5A2 expression of different BPH tissues and cells. (A) Immunohistochemical staining showed that the expression of SRD5A2 was varied in different BPH tissues. Sample 1: negative SRD5A2 expression. Sample 2: weak SRD5A2 expression. Sample 3: strong SRD5A2 expression. No antibody: negative control without incubation with a primary antibody. (B) Varied SRD5A2 expression in BPH tissues. (C)Different SRD5A2 expression in BPH-1 and RWPE-1 cells. Scale bars = 50 μm; ^***^p ≤ 0.001.SRD5A2: 5-α reductase type 2; BPH: benign prostate hyperplasia; BPH-1: immortalized benign prostatic hyperplasia epithelial cells; RWPE-1: normal prostate epithelial cell line. The original blots/gels are presented in Additional 1: Fig S1

To evaluate SRD5A2 expression in different prostate tissues, a total of 59 BPH specimens were collected. Immunohistochemical staining showed that SRD5A2 was expressed primarily in epithelial cells of the prostate transition zone, and a small amount of SRD5A2 was expressed in stromal cells, which was consistent with previous studies 6, 7, 18. In our study, SRD5A2 expression varied in different patients. Eight cases (13.6%) of BPH showed negative SRD5A2 expression, 17 cases (28.8%) showed weak expression, and 34 cases (57.6%) showed strong positive expression (Fig. 1 A, B). SRD5A2 expression in BPH-1 and RWPE-1, two classical cell lines of prostatic epithelium, were detected by western blotting. We found that SRD5A2 was weekly expressed in BPH-1, whereas strongly expressed in RWPE-1 cells (Fig. 1 C).

### miR-1199-5p is expressed differentially in different BPH tissues and cells

**Fig. 2:**
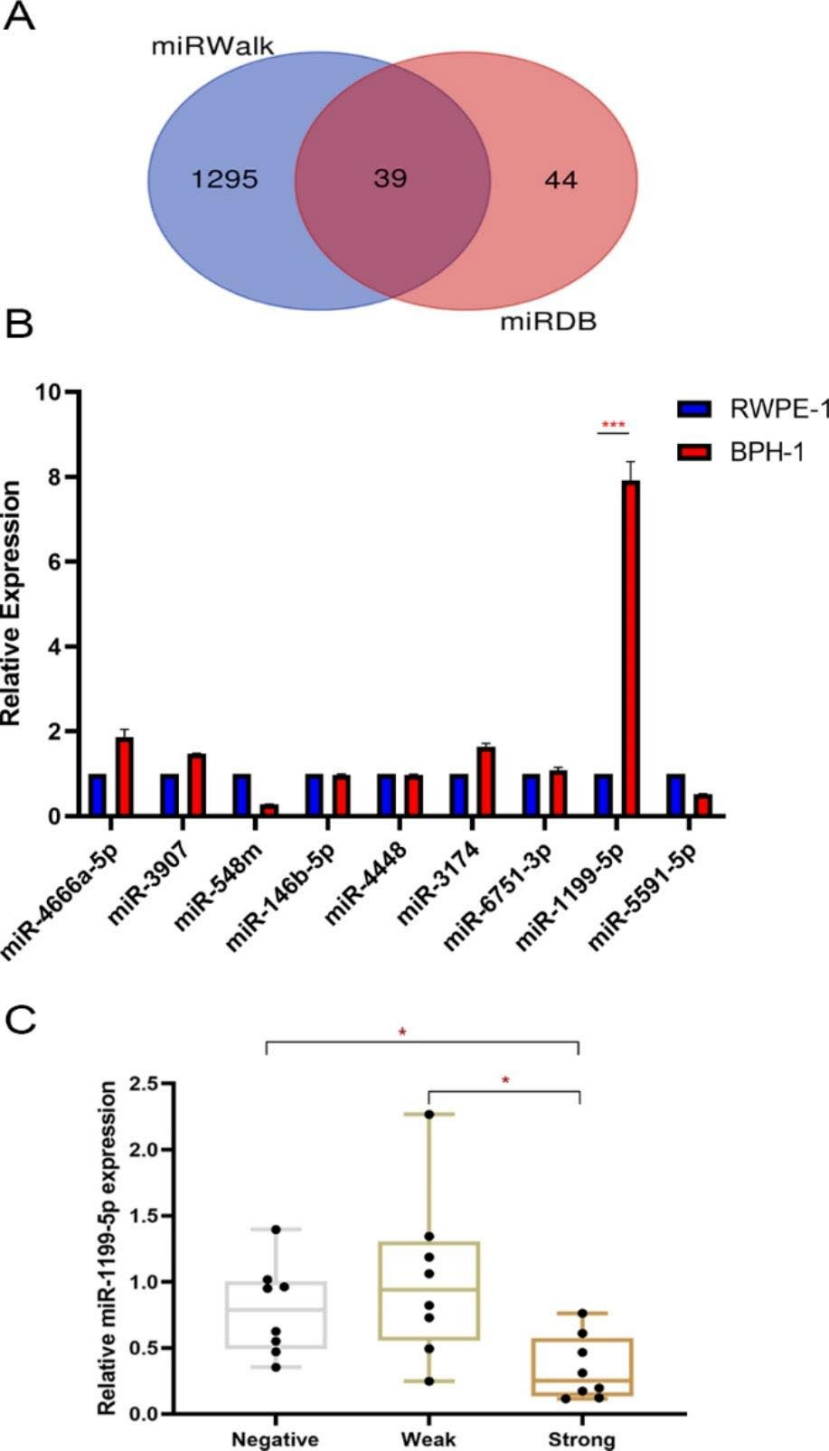
Prediction and screening of miRNA. (A) Venn diagram showed that 39 miRNAs were predicted to binding 3’UTR of SRD5A2 by miRWalk (http://mirwalk.umm.uni-heidelberg.de/) and miRDB (http://mirdb.org) database. (B)The RT-qPCR results showed that only the expression of miR-1199-5p in BPH-1 was significantly higher than that in RWPE-1. (C) The relative expression of miR-1199-5p in tissues with negative SRD5A2 expression, weak SRD5A2 expression, and positive SRD5A2. ^*^P<0.05;^***^P<0.001;miRNA: microRNA; SRD5A2: 5-α reductase type 2; BPH: benign prostate hyperplasia; BPH-1: immortalized benign prostatic hyperplasia epithelial cells; RWPE-1: normal prostate epithelial cell line

We used two databases, miRWalk 19 and miRDB 20, to predict potential miRNAs that might bind the 3′UTR of SRD5A2 mRNA. miRWalk predicted 1334 and miRDB predicted 83 potential miRNAs, with a total of 39 intersections (Fig. 2 A). We selected nine miRNAs, namely: miR-4666a-5p, miR-3907, miR-548 m, miR-146b-5p, miR-4448, miR-3174, miR-6751-3p, miR-1199-5p, and miR-5591-5p, from the 39 intersections with high prediction scores in the miRDB database as candidate miRNAs (Table S2).

To identify miRNAs that could affect SRD5A2 expression, we determined the relative expression of the nine candidate miRNAs in BPH-1 and RWPE-1 through qRT-PCR. We found that miR-1199-5p expression was significantly higher in BPH-1 than in RWPE-1 (Fig. 2B). To understand the interrelation between miRNAs and SRD5A2 expression in clinical samples, we analyzed the nine miRNAs expression in tissues with negative SRD5A2 expression, weak SRD5A2 expression, and positive SRD5A2 using qRT-PCR, and found that miR-1199-5p expression was significantly upregulated in tissues with negative and weak SRD5A2 expression than in those with positive SRD5A2 expression (Fig. 2 C). miR-4666a-5p, miR-3907, and miR-3174 expression were also higher in BPH-1 than in RWPE-1. But the three microRNAs expression had no difference in BPH tissues with different SRD5A2 expression (Fig S2).

### miR-1199-5p regulated SRD5A2 expression and suppressed the apoptosis of RWPE-1 cells

**Fig. 3:**
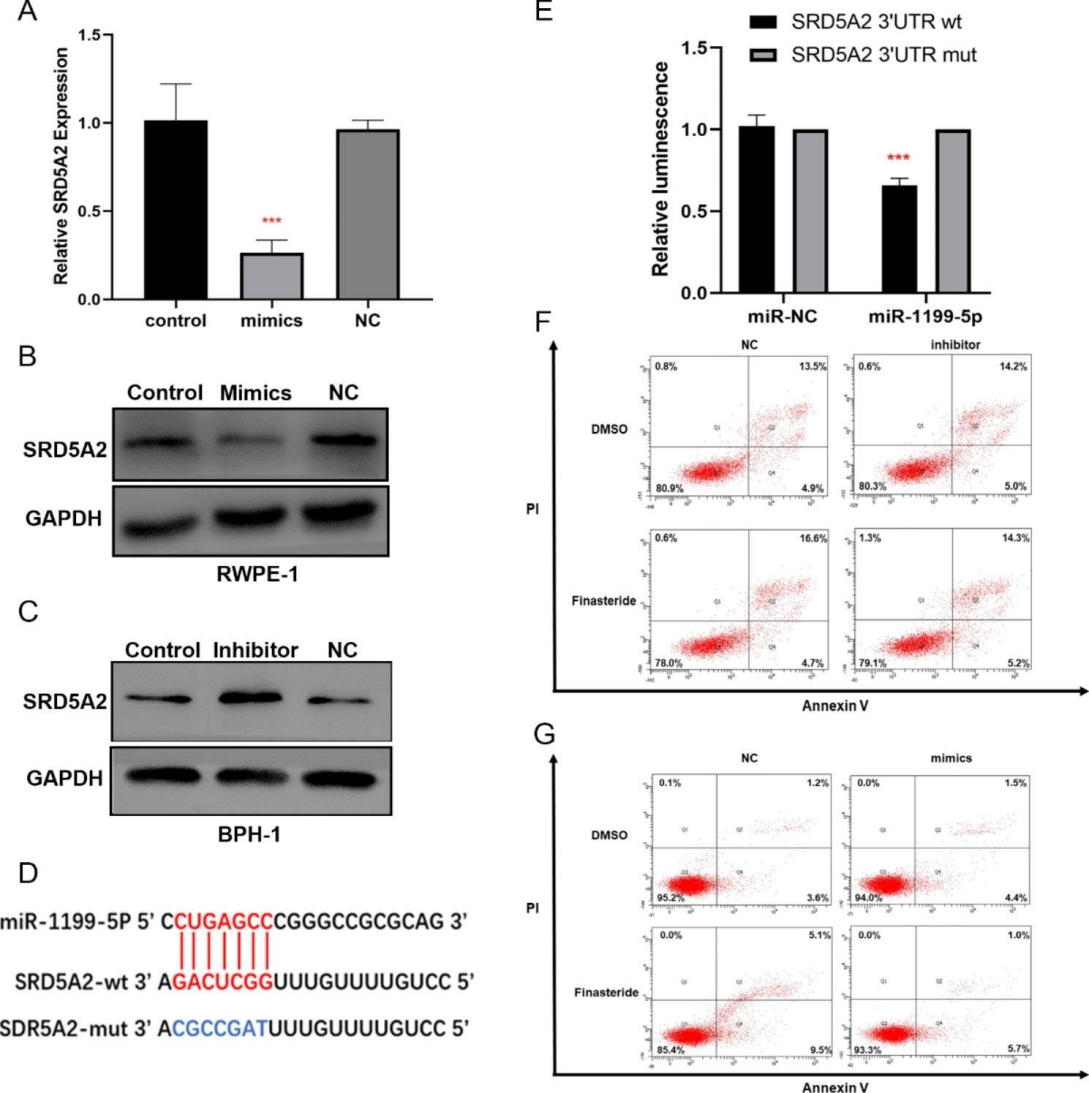
miR-1199-5p regulating the expression of SRD5A2. (A)The RT-qPCR results showed that SRD5A2 mRNA was significantly downregulated after transfection with miR-1199-5p mimics in RWPE-1 cells. (B-C) The Western Blotting results showed that miR-1199-5p mimics decreased the expression of SRD5A2 in RWPE-1 cells and miR-1199-5p inhibitors rescued the expression of SRD5A2 in BPH-1 cells. (D) Schematic representation of the mature miR-1199-5p sequence, putative miR‐1199-5p target site in the 3’UTR of SRD5A2 mRNA. (E) Overexpression of miR‐1199-5p markedly decreased the relative luciferase activity in the WT 3’UTR of SRD5A2 mRNA, while the mutated 3’UTR of SRD5A2 was insensitive to miR‐1199-5p overexpression. (F) The apoptosis of BPH-1 treated with finasteride (100 µM)/DMSO after transfection with miR-1199-5p inhibitor/ inhibitor-NC. (G) The apoptosis of RWPE-1 treated with finasteride (100 µM)/DMSO after transfection with miR-1199-5p mimics/mimics-NC. ***P<0.001; SRD5A2: 5-α reductase type 2; BPH-1: immortalized benign prostatic hyperplasia epithelial cells; RWPE-1: normal prostate epithelial cell line. The original blots/gels are presented in Additional file : Fig S3-4

To verify whether miR-1199-5p could regulate SRD5A2 expression, we transfected miR-1199-5p mimics into RWPE-1 cells that strongly expressed SRD5A2 and miR-1199-5p inhibitors into BPH-1 cells that weakly expressed SRD5A2. qRT-PCR and western blotting were used to detect the changes in SRD5A2 mRNA and protein expression, respectively, levels after transfection. We found that the mRNA (Fig. 3 A) and protein expression of SRD5A2 (Fig. 3B) were significantly decreased after RWPE-1 cells were transfected with miR-1199-5p mimics. Whereas the protein expression of SRD5A2 was significantly elevated in BPH-1 cells after transfection with miR-1199-5p inhibitor (Fig. 3 C). Additionally, we used the RNAhybrid (https://bibiserv.cebitec.uni-bielefeld.de/rnahybrid/) 21 to predict the binding sites of miR-1199-5p to SRD5A2. According to the specific binding sites, we constructed two SRD5A2 3′UTR luciferase reporter constructs—one containing the species conserved seed sequence and the other carrying a mutated version of the seed sequence with seven nucleotides exchanged (Fig. 3D). Transient transfection of miR-1199-5p mimics and the SRD5A2 3′UTR wild-type reporters in 293T cells revealed a significant decrease in luminescence, which was not observed in the mutant version of the reporter (Fig. 3E). These results confirmed that miR-1199-5p can bind to SRD5A2 3′UTR and inhibit its expression.

We also detected the influence of finasteride on the viability of BPH-1 and RWPE-1 after the transfection of miR-1199-5p mimics and inhibitors by flow cytometry. As we previously reported, finasteride did not induce apoptosis of BPH-1, which showed decreased SRD5A2 expression 9. However, increasing SRD5A2 expression in BPH-1 cells through miR-1199-5p inhibitor transfection did not lead to the development of finasteride (100 µM) sensitivity in BPH-1 cells (Fig. 3 F). Notably, finasteride (100 µM) promoted apoptosis of RWPE-1 and suppressed SRD5A2 expression through miR-1199-5p mimics transfection inhibited apoptosis progression (Fig. 3G). Different AR expressions might explain this phenomenon. Particularly, RWPE-1 cells express AR, whereas BPH-1 cells lack AR 22, 23.Thus, these data indicated that miR-1199-5p could decrease SRD5A2 expression and influence prostate cells apoptosis.

## Discussion

5-α reductase is the key enzyme in transforming T into DHT, the major steroid hormone that binds the AR, and SRD5A2 is the main subtype of SRD5A distributed in prostate tissues. BPH, androgenic alopecia, and prostate cancer are associated with high levels of DHT produced by SRD5A2 because of excessive AR signaling. Currently, 5-ARI therapy is one of the main medical treatments used to manage BPH and works by inhibiting SRD5A2 and subsequent conversion of T into DHT 24. Additionally, some medicinal herbs are used for BPH treatment targeting SRD5A2. Jin BR et al. found that baicalin ameliorated pathological prostate enlargement by inhibiting SRD5A2 activity and androgen-dependent apoptosis 22. Song KH et al. reported that extracts of Phyllostachys pubescens leaves repressed SRD5A2 promoter activity and enhanced BPH in a rat model. SRD5A2 expression varies in BPH tissues. We observed that 27% of BPH tissues showed weak SRD5A2 expression and 13.6% of tissues showed nearly no SRD5A2 expression, which is close to that reported by Lin(10%)^9^, and lower than that reported by Niu 6 and Wang(30%)^25^. Kang et al. suggested that the reduction of SRD5A2 in prostate tissue in some patients may be responsible for 5-ARI treatment failure 8. Understanding the reasons behind reduced SRD5A2 in prostate tissues may help identify patients resistant to 5-ARI treatment and further avoid ineffective treatments with adverse effects.

DHT has indispensable roles in the formation of male genitalia, prostate, urethra, and development of secondary sexual characters during puberty. Small prostate and female pseudohermaphroditism occur in human males that lack SRD5A2 expression during fetal period 8, 26. Therefore, epigenetic modifications that suppressed SRD5A2 expression during adulthood should account for the decreased SRD5A2 expression in some BPH patients. Epigenetic modifications, including DNA methylation, histone modifications, and miRNA modifications, induce reversible and heritable changes that alter gene expression without altering DNA sequence 27. The variability in SRD5A2 expression has been studied before. Austin DC et al. found that inflammation could promote SRD5A2 expression by activating NF-κB, resulting in BPH progression and 5-ARIs resistance 7. However, Ge R et al. reported that inflammatory mediators could decrease SRD5A2 expression by increasing the expression of DNMT1, that methylates the SRD5A2 promoter 12. Xue B et al. suggested that inflammatory mediators and saturated fatty acid could facilitate SRD5A2 promoter methylation by stimulating macrophages 11. Previous studies have shown that the methylation of the promoter region of SRD5A2 is closely related to the decrease in SRD5A2 protein expression and that age and inflammatory mediators are associated with high SRD5A2 promoter methylation 6, 8, 9, 11, 12. However, 10% of BPH tissues with negative SRD5A2 expression showed approximately no methylation in the promoter region of SRD5A2^9^, suggesting the existence of other mechanisms regulating SRD5A2 expression in the prostate.

miRNAs are small non-coding RNA widely expressed in various cells and bind to the 3′UTR of SRD5A2 mRNAs to form a silencing complex, causing mRNA degradation or translation repression 28. The role of miRNAs in prostate cancer has been widely reported, however, only a few have studied miRNAs in BPH. Zhang et al. identified 26 dysregulated miRNAs between patients with BPH and healthy men and suggested that aberrantly expressed miRNAs may be involved in BPH development 29. Wang et al. reported that the long noncoding RNA RNM3OS inhibited miR-361 and miR-29a/29b, which suppressed the expression of COL3A1 and TGF-β1, leading to BPH development by promoting TGFβ1-induced transformation of prostate stromal cells into myofibroblasts 30. However, there are no relevant studies on miRNA and SRD5A2. In our study, we found that miR-1199-5p expression was significantly higher in BPH tissues with negative and weak SRD5A2 expression than those with positive SRD5A2 expression. Additionally, SRD5A2 expression decreased in RWPE-1 transfected with miR-1199-5p mimics, whereas SRD5A2 expression was rescued in BPH-1 cells transfected with miR-1199-5p inhibitors. We further verified, through luciferase assay, that miR-1199-5p regulated SRD5A2 expression by binding the 3′UTR of SRD5A2.

After SRD5A2 expression decreased, the mechanisms promoting BPH were still under discussion. We found that decreased SRD5A2 expression in RWPE-1 could increase the insensitive to finasteride (100 µM) treatment after miR-1199-5p mimics transfection. Besides, Wang et al. reported an androgen to estrogen switch in the absence of SRD5A2 resulting from promoter methylation in prostate tissues, followed by estrogen playing the dual role of proliferation or inhibition via ERα or ERβ 25. Although testicles secrete small amounts of estrogen, 75–90% of circulating estrogens in men are converted by the action of aromatase 31. Chen et al. identified that estrogen promoted the proliferation of primary stromal cells and increased CCR3, CD40L, CXCL9, IL-10, and IL-17 in BPH tissues 32. We detected estrogen levels in ten pairs of prostate tissues with weak or strong SRD5A2 expression and found that estrogen levels were significantly higher in prostate tissues with weak SRD5A2 expression than in those with strong SRD5A2 expression (P = 0.0002, the result is not shown), suggesting that SRD5A2 was inversely related to estrogen in prostate tissue.

To the best of our knowledge, our study is the first to explore the correlation between miRNA and SRD5A2 expression. miR-1199-5p may serve as a marker to screen patients with different SRD5A2 expression levels in clinical settings and facilitate personalized treatment. There are a few limitations to our study. Due to the limited number of tissues, there was no definite difference in miR-1199-5p expression between tissues with negatively and weakly expressed SRD5A2. Thus, further research with a large sample size of BPH tissues is required. Besides, the BPH tissues collected in this study did not exclude patients treated with 5-ARIs before surgery; hence, it was unclear whether 5-ARIs could affect SRD5A2 expression. Therefore, further research is needed to support our hypothesis that miR-1199-5p interferes with SRD5A2 expression and its clinical implications in personalized therapy for BPH.

## Electronic supplementary material

Below is the link to the electronic supplementary material.

Supplementary Material 1

Supplementary Material 2

Supplementary Material 3

Supplementary Material 4

Supplementary Material 5

Supplementary Material 6

Supplementary Material 7

## Data Availability

All data generated or analyzed during this study are available on reasonable request from the corresponding author.

## Abbreviations

**5-ARIs**: 5-α reductase inhibitors
**BPH**: benign prostatic hyperplasia
**SRD5A2**: 5-α reductase type 2
**LUTSs**: lower urinary tract symptoms
**T**: testosterone
**DHT**: dihydrotestosterone
**ARs**: androgen receptors
**EMT**: epithelial-mesenchymal transition
**IHC**: Immunohistochemistry
**FBS**: fetal bovine serum
**qRT-PCR**: quantitative real-time PCR
**ECL**: Enhanced chemiluminescence
