# Supplementary figures and images for: Upregulation of mir-1199-5p is associated with reduced type 2 5-α reductase expression in benign prostatic hyperplasia
Source: BMC Urol. 2022 Nov 7;22:172. doi: 10.1186/s12894-022-01121-5 (PMC9639318; doi:10.1186/s12894-022-01121-5)

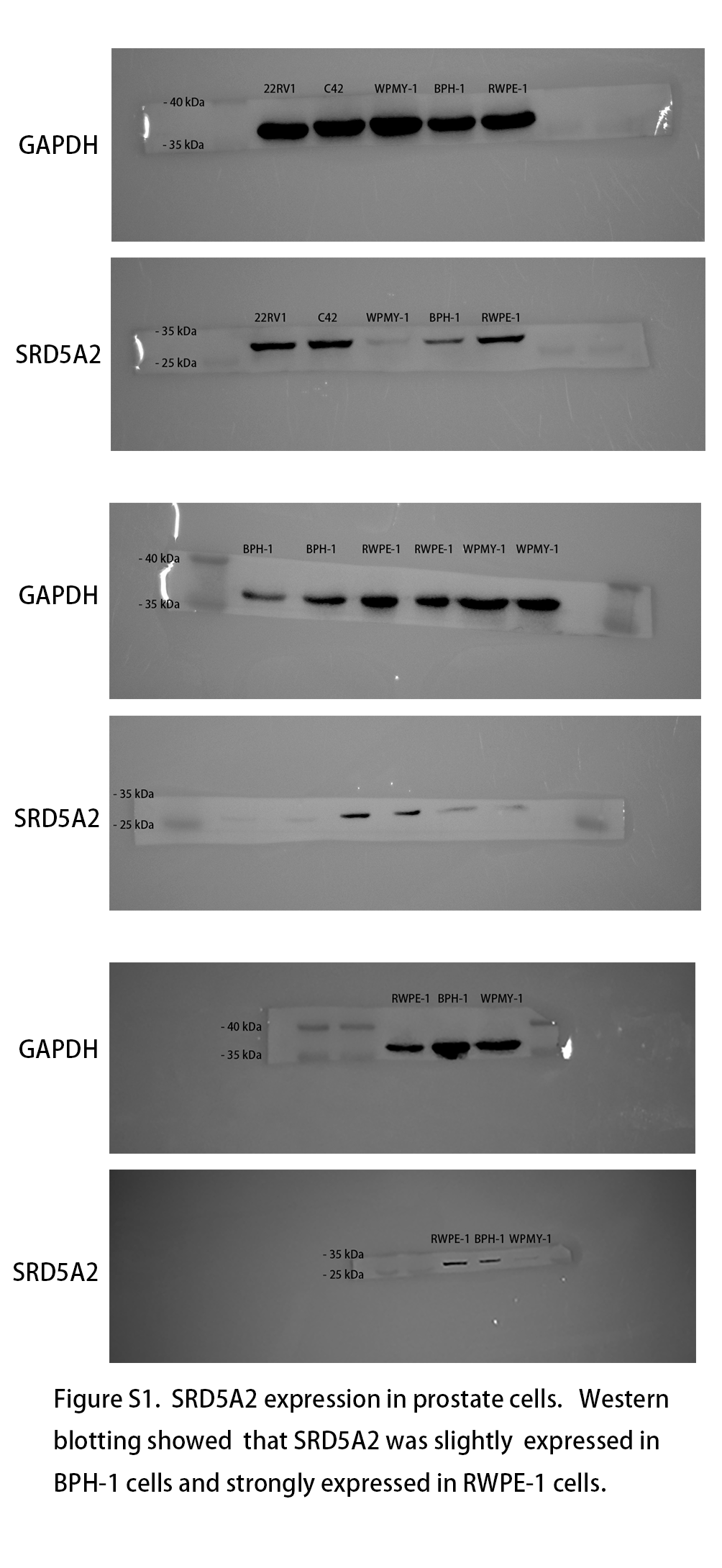

Supplement: Supplementary file 1 — Supplementary Material 1 [file 12894_2022_1121_MOESM1_ESM.png]

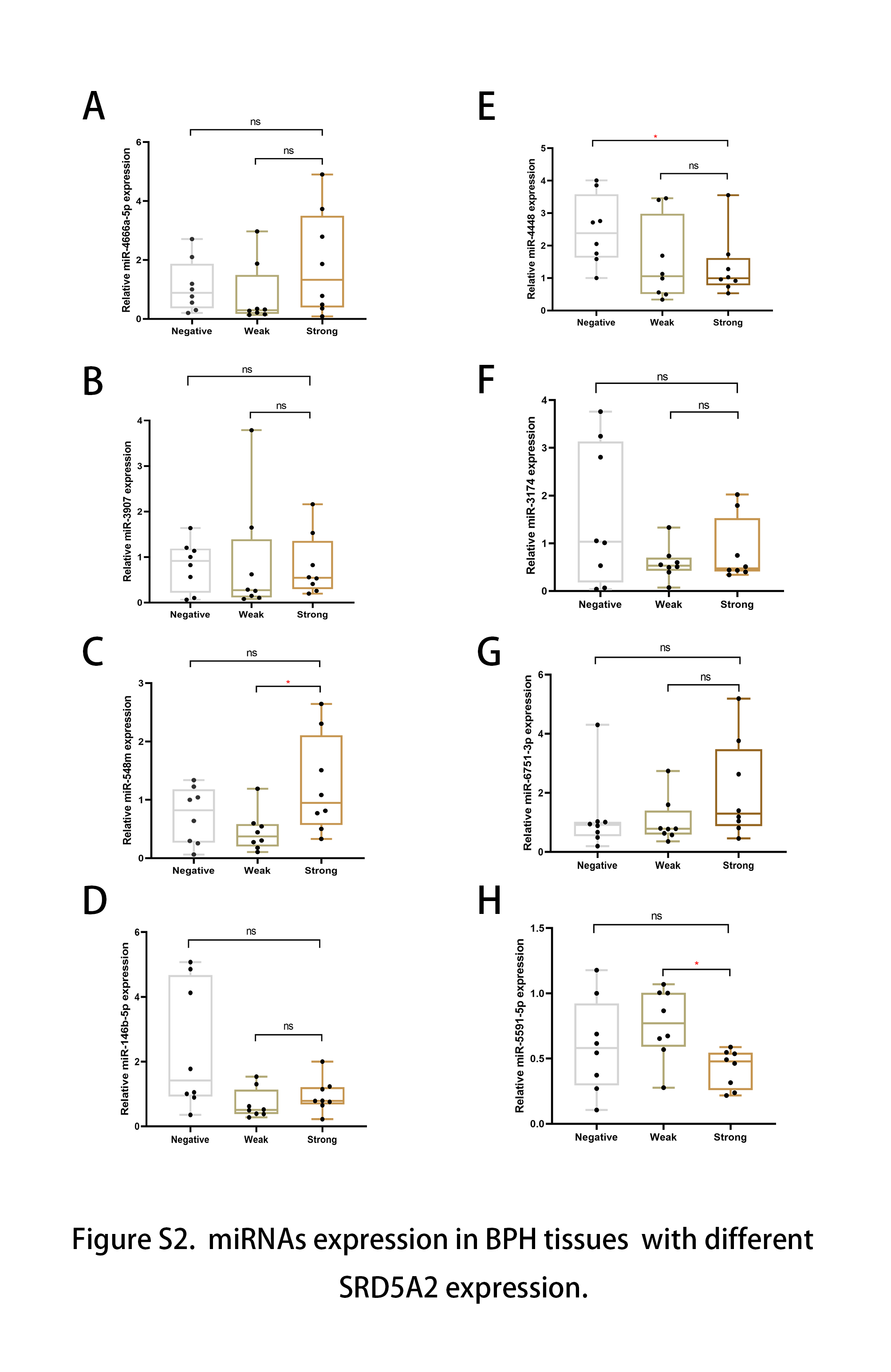

Supplement: Supplementary file 2 — Supplementary Material 2 [file 12894_2022_1121_MOESM2_ESM.png]

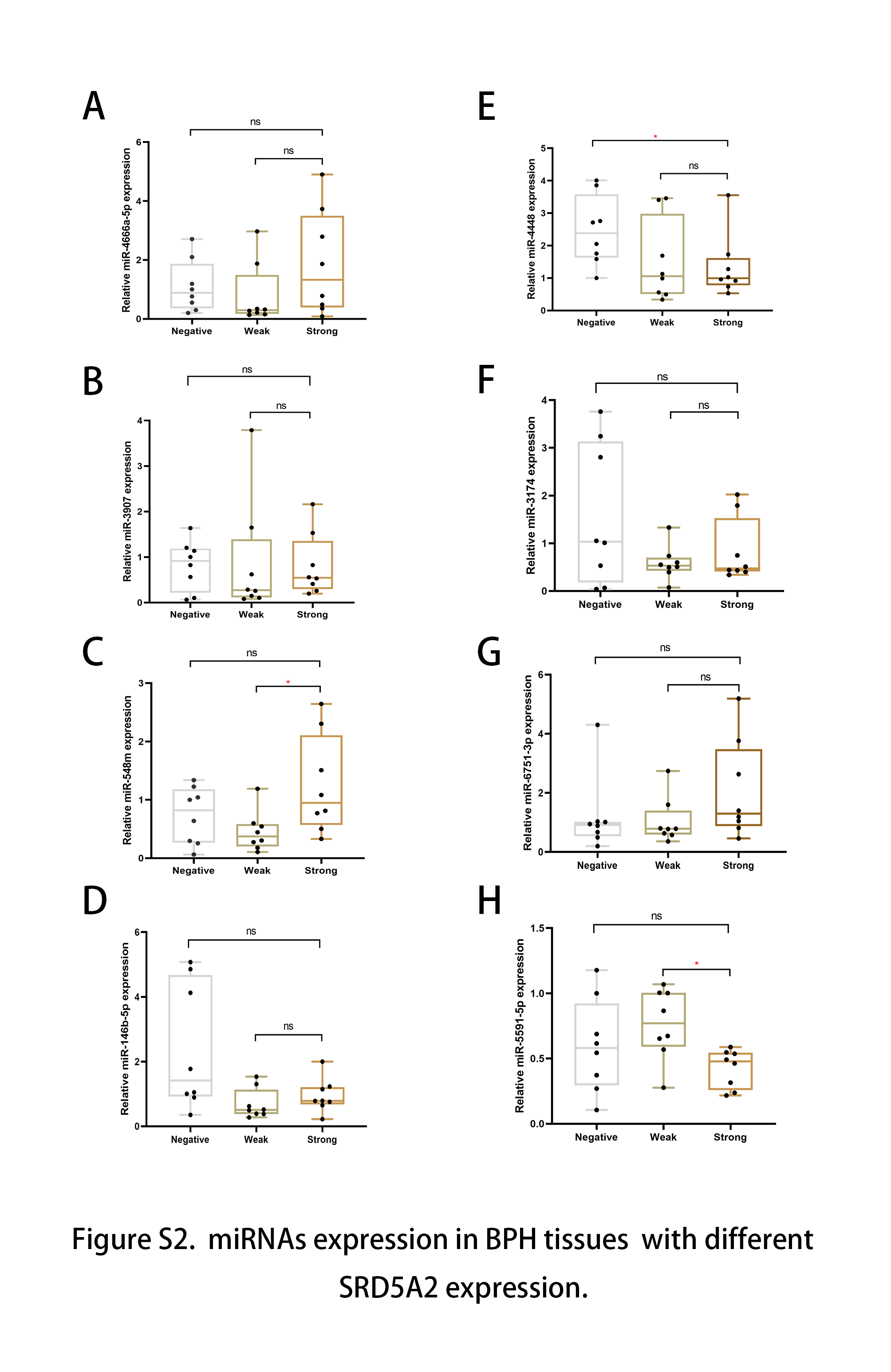

Supplement: Supplementary file 3 — Supplementary Material 3 [file 12894_2022_1121_MOESM3_ESM.png]

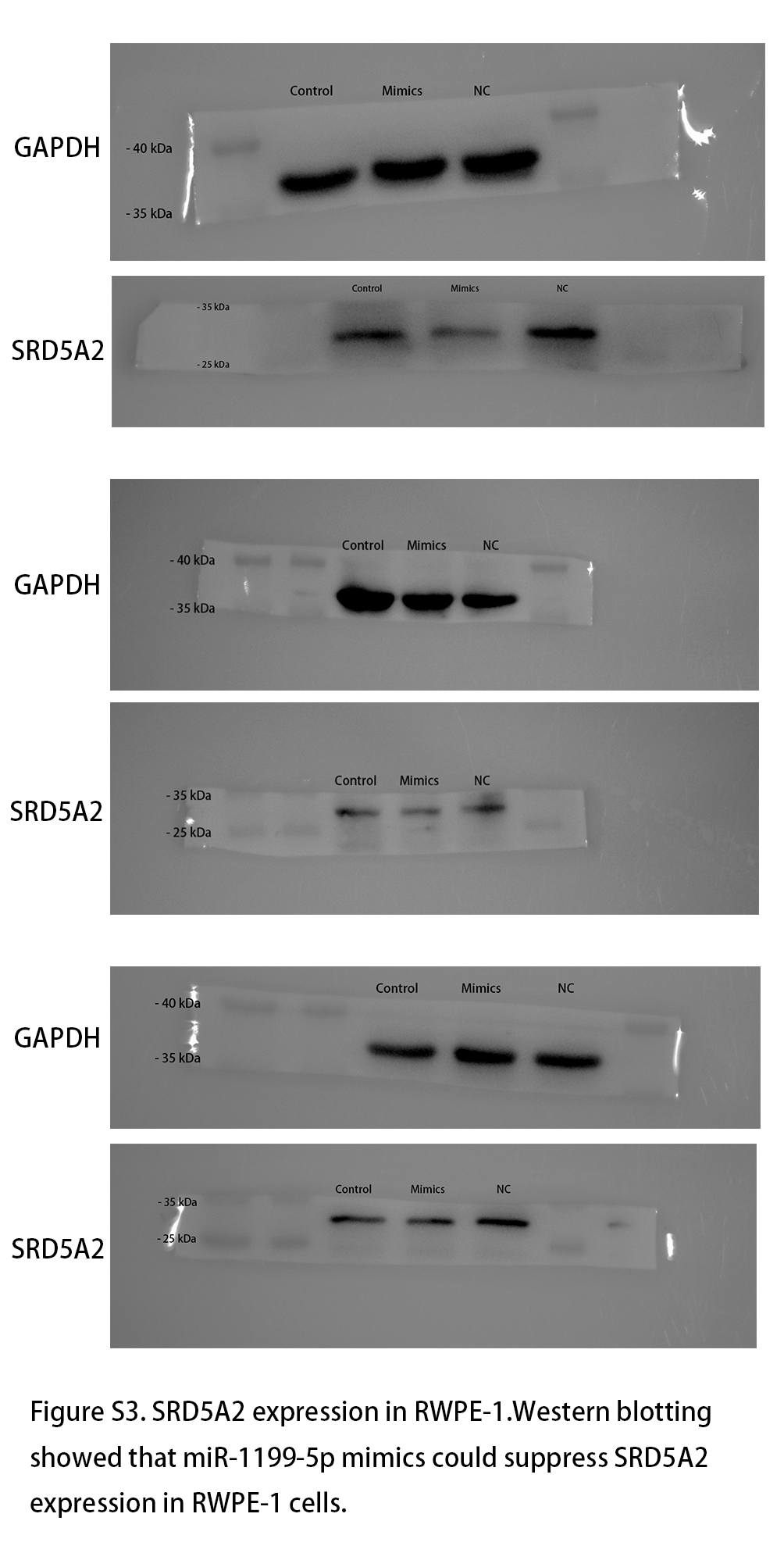

Supplement: Supplementary file 4 — Supplementary Material 4 [file 12894_2022_1121_MOESM4_ESM.png]

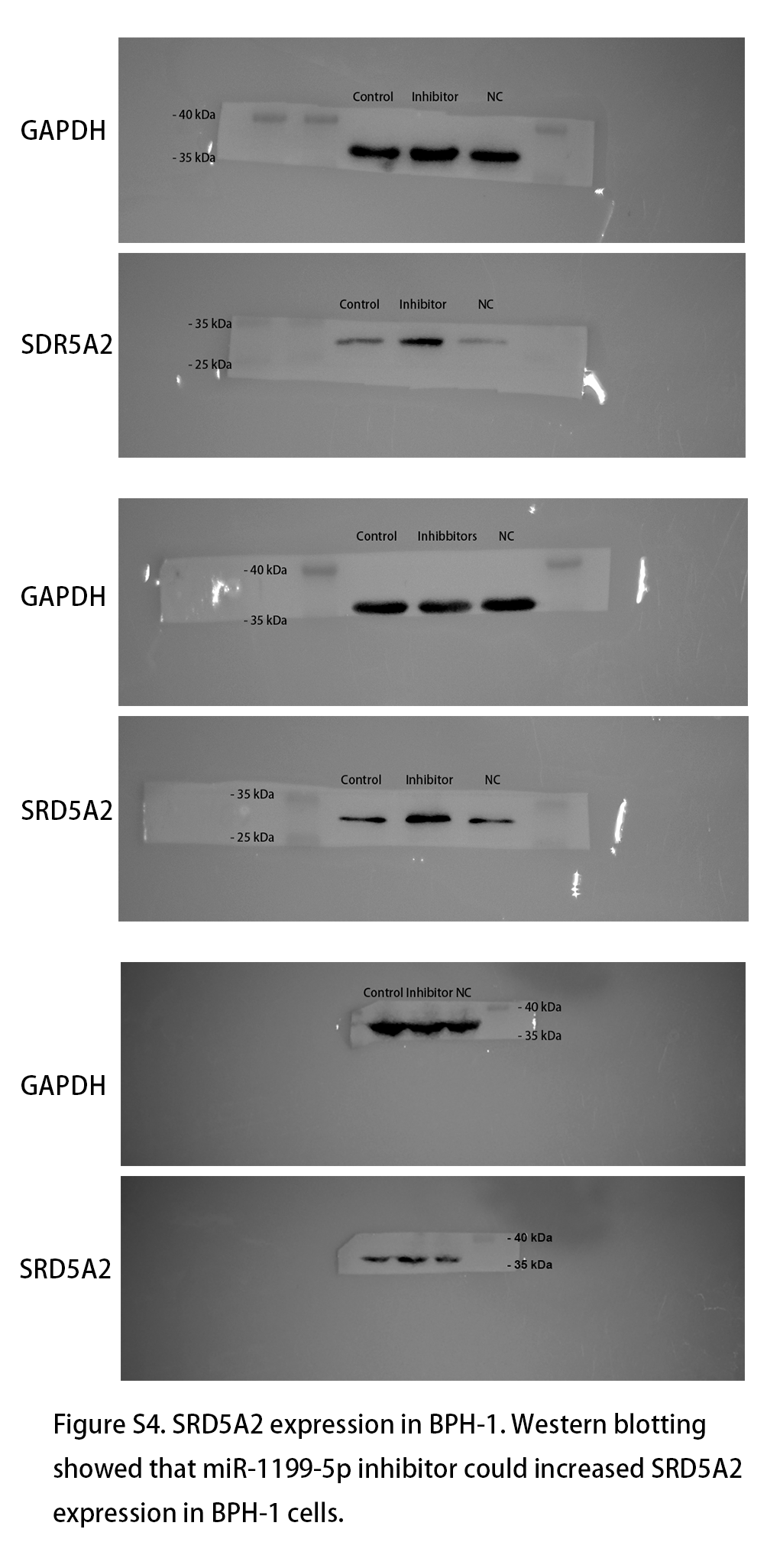

Supplement: Supplementary file 5 — Supplementary Material 5 [file 12894_2022_1121_MOESM5_ESM.png]
