# Supplementary material for: Upregulation of mir-1199-5p is associated with reduced type 2 5-α reductase expression in benign prostatic hyperplasia
Source: BMC Urol. 2022 Nov 7;22:172. doi: 10.1186/s12894-022-01121-5 (PMC9639318; doi:10.1186/s12894-022-01121-5)
Supplement: Supplementary file 6 — Supplementary Material 6 [file 12894_2022_1121_MOESM6_ESM.docx]

| Gene | Direction | Primer sequence (5'- 3') |
| --- | --- | --- |
| miR-4666a-5p | F | CGCGATACATGTCAGATTGTATGCC |
| miR-3907 | F | TATATAGGTGCTCCAGGCTGGC |
| miR-548m | F | CGCGCAAAGGTATTTGTGG |
| miR-146b-5p | F | GTCCAGTTTTCCCAGGAATCCC |
| miR-4448a | F | GGCTCCTTGGTCTAGGGGTA |
| miR-3174 | F | CCTAGTGAGTTAGAGATGCAGAGCC |
| miR-6751-3p | F | ACTGAGCCTCTCTCTCTCCAG |
| miR-1199-5p | F | TATATATACCTGAGCCCGGGCC |
| miR-5591-5p | F | GATGCCCATGCCGATTCTT |
| U6 | F | CTCGCTTCGGCAGCACA |
|  | R | AACGCTTCACGAATTTGCGT |
| SRD5A2 | F | ACTGCTCAATCGAGGGAGG |
|  | R | CACCCAAGCTAAACCGTATGTC |
| GAPDH | F | GAACGGGAAGCTCACTGG |
|  | R | GCCTGCTTCACCACCTTCT |
| β-actin | F | GAGCGGGAAATCGTGCGTGACATT |
|  | R | GATGGAGTTGAAGGTAGTTTCGTG |

Table2. Primer sequences used in reverse transcription quantitative-polymerase chain reaction
