# Supplementary material for: Upregulation of mir-1199-5p is associated with reduced type 2 5-α reductase expression in benign prostatic hyperplasia
Source: BMC Urol. 2022 Nov 7;22:172. doi: 10.1186/s12894-022-01121-5 (PMC9639318; doi:10.1186/s12894-022-01121-5)
Supplement: Supplementary file 7 — Supplementary Material 7 [file 12894_2022_1121_MOESM7_ESM.docx]

| miRNA | Prediction score |
| --- | --- |
| miR-4666a-5p  miR-3907  miR-548m  miR-146b-5p  miR-4448  miR-3174  miR-6751-3p  miR-1199-5p  miR-5591-5p | 90  87  85  81  78  77  75  75  73 |

Table3. Prediction scores of miRNA
